# Investigating differences in common mental health symptom expression and co-occurrence across ethnicities

**DOI:** 10.1186/s44263-026-00274-x

**Published:** 2026-05-01

**Authors:** Henry Delamain, Jonas Haslbeck, Madiha Shaikh, Joshua Eusty Jonathan Buckman, Renuka Jena, Rob Saunders, Joshua Stott, Jae Won Suh, Stephen Pilling, Ciarán O’Driscoll

**Affiliations:** 1 CORE Data Lab, Centre for Outcomes Research and Effectiveness (CORE), Research Department of Clinical, Educational and Health Psychology, University College London, 1-19 Torrington Place, London, WC1E 7HB UK; 2 Department of Psychological Methods, University of Amsterdam, Amsterdam, Netherlands; 3 Department of Clinical Psychological Science, Maastricht University, Maastricht, Netherlands; 4 Research Department of Clinical, Educational and Health Psychology, University College London, London, UK; 5 iCope - Camden and Islington Psychological Therapies Services - Camden & Islington National Health Service Foundation Trust, London, UK; 6 Waltham Forest Talking Therapies, North East London NHS Foundation Trust, London, UK; 7 Adapt Lab, Research Department of Clinical, Educational and Health Psychology, University College London, London, UK; 8 Camden and Islington NHS Foundation Trust, London, UK

**Keywords:** Ethnic inequalities, Mental disorders, Moderated network analysis

## Abstract

**Background:**

Ethnic inequalities exist in the prevalence of mental disorders and their associated treatment outcomes. Cultural variation may influence psychological symptom expression; understanding this might inform care and ultimately reduce care disparities.

**Methods:**

Data were analysed from 147,037 individuals referred to psychological treatment services in London, England. Moderated network analysis was used to examine the expression and co-occurrence of symptoms of depression, anxiety, and social functioning. Age and gender were included as covariates, and ethnicity (11 categories) was entered as the moderating variable. Symptom-level differences and differences in symptom-to-symptom relationships were estimated across all ethnic group comparisons.

**Results:**

There was substantial variation in symptom networks between ethnic groups. White British individuals showed the most differences compared to other ethnic groups, particularly with higher endorsement of anxiety-related symptoms (e.g. nervousness and excessive worry) and greater reported functional impairment in work and social domains. Differences in symptom co-occurrence (the relationships between symptoms) were less frequent than differences in individual symptom levels. Across groups, several symptoms showed consistent relationships, suggesting shared aspects of distress alongside culturally patterned variation in symptom expression.

**Conclusions:**

The influence of ethnicity on both symptom levels and symptom co-occurrence underscores the importance of culturally informed assessment. These findings highlight the need for services to consider culturally relevant symptom presentations to promote more equitable and appropriate mental health care.

**Supplementary Information:**

The online version contains supplementary material available at 10.1186/s44263-026-00274-x.

## Background

Mental disorders are among the leading causes of global disability, with depression and anxiety rates rising across all world regions [1] at an estimated global cost of 1 trillion United States (US) dollars per year [2]. How individuals experience anxiety and depression varies across populations and cultures; this challenges the universality of current diagnostic frameworks [3]. Substantial differences in the perception and expression of mental health symptoms across the world have been reported globally [4, 5]. Studies across cultures have found common depression symptoms not included in Diagnostic and Statistical Manual of Mental Disorders, Fifth Edition (DSM-5) criteria, including loneliness, crying, anger, and pain, with sadness being most reported in Western and African populations, and fatigue in Asian populations [5]. This study therefore examines how commonly used DSM-derived measures capture symptom expression across ethnic groups, to identify where standard symptom measures may not fully reflect variation in experiences of distress across groups. Despite recognition of these differences, mental health care remains largely based on Western constructs [6], potentially contributing to persistent ethnic inequalities in treatment access and outcomes [7].

In the United Kingdom (UK), significant barriers to the access of mental health services persist, particularly in initial access to care. People from minoritised ethnic groups are less likely to self-refer to psychological services and face higher rates of declining mental health assessment, with Black African communities showing the highest rates of declining assessments [8] and delayed interventions [9]. Immigration status adds another layer of complexity, with recent migrants having lower rates of general practitioner (GP) referrals and service attendance compared to longer-term residents [10]. The UK-specific findings highlight that, even within a single national healthcare system, ethnic disparities in mental health outcomes remain evident.

Recovery outcome rates for psychotherapy are lower for most ethnic groups compared to White British patients [11]. This disparity is particularly pronounced among Asian, Other, and White Other groups [12]. These labels reflect the ethnic classifications used within the original UK-based studies and may differ from terminology employed in other countries’ health systems. The categories include multiple subgroups (e.g. Indian, Pakistani, Bangladeshi within Asian; Chinese and other backgrounds within ‘Other’; and non-British White backgrounds within ‘White Other’), as defined by the UK Office for National Statistics (ONS). Such access patterns highlight the value of examining symptom expression across ethnic groups, given that variations in presentation may then contribute to inequalities observed across the care pathway. Research indicates a complex relationship, showing that among young adults who are not in employment, education, or training (NEET), those from minoritised ethnic groups had higher attendance rates and were more likely to achieve reliable recovery than their White counterparts [13]. The NEET finding reflects a specific subgroup whose engagement patterns differ from the broader population and therefore does not contradict the overall trend of lower recovery rates among minoritised groups in routine care. These disparities have been attributed to multiple factors including: differences in mental health awareness, cultural perspectives, immigration status, stigma, and social isolation [14]. The role of culturally specific symptom expression in these disparities remains poorly understood. Understanding how psychological symptoms manifest and interact across different ethnic groups could therefore provide insights for developing more culturally informed mental health care.

Culture encompasses the learned and shared behaviours, meanings, and adaptations that help individuals fit into their environment [15]. It shapes our values, attitudes, beliefs, thought processes, worldviews, and concept of personhood [16]. In multicultural societies, culturally competent mental health services are increasingly vital [17]. Meta-analyses of cultural adaptations in psychotherapy have yielded both promising findings and concerns. Several studies suggest that culturally adapted interventions are more effective than non-adapted ones [18], particularly native language interventions [19]. Adaptations focus on implementation rather than content [20] and are more effective with additional adaptation elements, particularly when targeting specific cultural groups [19]. Therapists’ cultural competence has been linked to various therapeutic outcomes [21], though cultural competence training does not necessarily lead to greater skill [22]. Concerns remain about methodology, with little evidence of consistent improvements, low power and poor representation of less acculturated groups [23, 24]. Additionally, the cost-effectiveness of developing such interventions for smaller cultural groups remains debated [25]. This context highlights the importance of understanding how symptoms are expressed across groups, as cultural adaptations of care depend on accurately identifying which aspects of distress are most salient within different populations.

While many adaptations focus primarily on how therapies are implemented (such as the language, format, or setting), adapting the content itself is crucial to ensure that psychological constructs and symptom targets within therapy reflect culturally specific experiences of distress. To better inform and optimise cross-cultural adaptation of therapies, whilst focusing on content rather than implementation, it is important to understand cross-cultural differences in symptom presentation [26]. Examining how symptoms co-occur within different groups can indicate which symptom clusters are most relevant or central within a particular cultural context, helping identify culturally meaningful patterns of distress that can guide more targeted and relevant therapeutic adaptations. The lens of symptom co-occurrence, where multiple symptoms or disorders present simultaneously, offers a promising avenue for such exploration [27]. Research exploring symptom co-occurrence within networks, reflecting the reality of how symptoms interact and influence each other, remains limited [28–30]. In this context, a network refers to a set of symptoms represented as nodes that are connected by statistical associations (edges), indicating how strongly symptoms relate to one another after accounting for all other symptoms. Network dynamics describe how these patterns of connections vary across groups, reflecting differences in how symptoms cluster or interact. Specifically, only one small cross cultural comparison study (*n* = 874) has examined depression and anxiety symptoms across three comparison groups/countries [31]. Our understanding of how symptom networks may vary across multiple ethnic groups within a single diverse society remains largely unexplored. This highlights a significant gap, as symptom network dynamics among multiple ethnic groups within the same country may differ considerably from cross-country comparisons, particularly in increasingly multicultural societies.

The objective of this study is to investigate differences in symptom presentation and co-occurrence across ethnicities among individuals seeking psychological treatment for anxiety and depression in the United Kingdom. The findings might inform more culturally sensitive care by improving the identification of disorders, treatment selection, and measuring patient progress. This approach promotes fairer and more equitable mental health support for all communities in multicultural societies.

## Methods

Differences in symptom presentation and co-occurrence were explored using broad ethnic categories defined by the ONS, achieved through analysing depression and anxiety symptom measures within a large sample of adults presenting at primary care psychological services. Populations in these categories are highly heterogeneous [32], encompassing variations in national identity, generational status, and immigration history [33]. The significance of these dimensions can differ among individuals. For instance, in the 1991 and 2001 UK Censuses, Black groups frequently highlighted their national identity, such as being British, as a key part of their ethnic identity [34]. In contrast, South Asian groups tended to emphasise religion as a crucial component of their ethnic identity [35, 36]. Therefore, these categories should be considered as proxies for cultural heritage rather than definitive cultural or ethnic groups [37]. While we recognise this heterogeneity, the ONS categories represent the standardised classification used within UK health datasets, allowing for consistent comparisons across services and making it possible to explore broad patterns in symptom expression at scale.

### Participants

Data were provided from patients presenting to eight National Health Service (NHS) Talking Therapies for anxiety and depression (NHS TTad; formerly known as Improving Access to Psychological Therapies, IAPT; https://www.england.nhs.uk/mental-health/adults/nhs-talking-therapies/) services between January 2011 and August 2020. The services are all members of the North and Central East London NHS TTad Service Improvement and Research Network [38] that support the stepped-care provision of evidence-based psychological therapeutic treatments for common mental disorders [39]. At the point of initial assessment, patients are asked to provide sociodemographic (e.g., age, ethnicity, and gender) and clinical information including mental health symptom scores. From the full sample of individuals presenting at assessment (*n* = 483,683), patients were excluded if they had any missing item-level assessment data for the psychological measures detailed below (*n* = 316,282), or missing demographic information (age, ethnicity and gender; *n* = 20,364).

### Measures

The Patient Health Questionnaire-9 (PHQ-9) [40] includes nine items that measure the degree of depression symptom severity within the last 2 weeks across the following items: anhedonia, low mood, sleep, fatigue, appetite, low self-esteem, concentration, psychomotor disturbance and suicidal ideation. The items are scored between 0 (‘not at all’) and 3 (‘nearly every day’), with total scores ranging between 0 and 27.

The Generalised Anxiety Disorder-7 (GAD-7) [41] includes seven items that measure the degree of generalised anxiety disorder symptom severity within the last 2 weeks across the following items: nervousness, uncontrollability of worrying, pervasiveness of worrying, issues relaxing, restlessness, irritability and anticipatory fear. The items are scored similarly to the PHQ-9, with total scores ranging between 0 and 21. 

The Work and Social Adjustment Scale (WSAS) [42] includes five items that measure the level of personal functioning (scored between 0 and 8) within the last week across the following items: ability to work, home management, social activities, private leisure activities and close relationships. The first item (‘ability to work’) is often recorded as not applicable for individuals not in employment and was not included in the analysis, in a similar manner to prior research [43, 44], as retaining it would have resulted in the exclusion of a substantial proportion of the sample. Total scores can range between 0 and 40. We included the WSAS to capture functional impairment alongside symptoms, as understanding how symptoms relate to day-to-day functioning is central to comparing clinical presentation across ethnic groups.

### Ethnicity

Ethnicity status is collected as part of the NHS TTad minimum dataset that is based on the categories defined by the Office of National Statistics [45]. There are five broad categories, with sub-categories that individuals can then select from within NHS TTad: White (British, Irish, Any other White background), Mixed/Multiple Ethnic Groups (White and Black Caribbean, White and Black African, White and Asian, Any other mixed background), Asian/Asian British (Indian, Pakistani, Bangladeshi, Any other Asian background), Black/African/Caribbean/Black British (Caribbean, African, Any other Black background) and Other Ethnic Groups (Chinese, Any other ethnic group); see Table 1 for further details on how the groups were categorised, including the original ONS categorisations.

**Table 1:** Office of National Statistics Ethnicity Categories

**White**	**Mixed/Multiple Ethnic Groups**	**Asian/Asian British**	**Black/African/Caribbean/Black British**
1 British	4 Mixed ethnicities (white/Asian, white/African, white/Caribbean, multiple ethnic background)	5 Indian	9 African
2 Irish		6 Pakistani	10 Caribbean
3 Any other White background		7 Bangladeshi	11 Any other Black/African/Caribbean background
		8 Asian	

Notes. Out of the sixteen ethnic groups available (‘Gypsy or Irish Traveller’ and ‘Arab’ did not exist within the dataset), the following groups were categorised to ensure sufficient sample size for comparison: Mixed ethnicity combined: ‘White and Black Caribbean’, ‘White and Black African’, ‘White/Asian’, and ‘Multiple ethnic background’, Asian-other-or-Chinese combined: ‘Chinese’ and ‘Any other Asian’ background, and ‘Any other ethnic group’ was removed

### Statistical analysis

We used Moderated Network Models (MNMs) to examine whether the structure of symptom networks differs across ethnic groups. This approach allows simultaneous estimation of symptom relationships while testing moderation by group membership, providing a framework for assessing differences in symptom expression and co-occurrence across groups. The network model included all symptom items from three measures as nodes: the PHQ-9 (depression), GAD-7 (anxiety), and WSAS (functional impairment, excluding item 1). Ethnicity served as the moderator variable, with models controlling for age and gender as routinely collected variables. In this context, the intercept represents the average level of a symptom for a given group after adjusting for these covariates and all other symptoms.

Differences in intercepts and partial associations were estimated in networks across all pairs of ethnicities. Partial associations between two symptoms differ from correlations because they account for the influence of all other symptoms. This makes them unique: they capture the direct relationship between two symptoms that cannot be explained by any other symptom in the network.

MNMs were estimated using the nodewise (or disjoint pseudolikelihood) approach, implemented in the R-package *mgm* [46]. This computational strategy simplifies estimation by calculating the individual contribution of each node separately rather than fitting the entire network simultaneously. Inference was performed with the Least Absolute Shrinkage and Selection Operator (LASSO) [47], a regularization technique that promotes sparse, interpretable models by penalising overly complex parameter estimates at the node level. This requires selecting a regularisation parameter (λ) that controls the strength of this penalty for each nodewise regression; this was determined using the Extended Bayesian Information Criterion (EBIC) [48].

Standard MNM implementations can estimate group differences and perform hypothesis tests when comparing multiple groups to a single reference group. However, the mgm package’s reliance on regularization for inference creates a limitation: it cannot directly estimate all pairwise group contrasts within a single model. To overcome this constraint and obtain all possible ethnic group comparisons, we adopted an iterative approach. We estimated multiple MNMs, systematically rotating which ethnic group served as the reference category in each model. This procedure generated the complete set of pairwise contrasts despite the single-reference-group restriction inherent to the implementation.

This iterative procedure produced two estimates for each group difference between any pair of ethnic groups (A and B): one from a model with group A as the reference, and another with group B as the reference. To synthesize these complementary estimates, we applied the AND-rule decision criterion [46]. Under this conservative rule, we concluded that a genuine group difference exists in the population only when both estimates were nonzero; if either estimate was zero (suggesting no difference), we concluded no group difference existed. This approach provides greater stability and reduces false positives under LASSO regularization compared to the alternative OR-rule (which would conclude a difference exists if either estimate is nonzero), as well as improving the robustness of detected group differences.

While inference was already performed on the group differences with the LASSO, the uncertainty associated with the detected group differences can be difficult to interpret. To address this and provide more standard statistical inference, we conducted permutation tests on all detected group differences. To that end, permutations of the original data were created across the groups and the 11 MNMs were refit on these data 100 times to obtain a sampling distribution under the null hypothesis that there are no group differences. The sampling distribution was then used to compute empirical *p*-values for group differences in individual parameters but also for aggregate parameters. Given the large number of related comparisons, results are interpreted at an aggregate level by comparing the number of observed significant differences to what would be expected under the null hypothesis, rather than applying formal multiple-comparison corrections to individual parameters. This combined strategy, using LASSO regularisation (which reduces the likelihood of spurious edges when estimating many parameters) followed by permutation-derived empirical *p*-values provides a pragmatic approach to handling multiple comparisons within this specific modelling framework. Due to the substantial sample size, 100 permutations were sufficient to approximate the null distribution for group differences, and the *mgm* package required refitting 11 MNMs per permutation to obtain the full set of pairwise ethnic group contrasts. This permutation procedure was used to provide empirical significance estimates for group differences and is not intended as a test of parameter stability.

All analyses used complete-case data; individuals with missing item-level responses or demographic information were excluded as previously described.

The results are presented at an aggregate level to provide an overview of group differences. For each pair of groups, two key comparisons are reported: (1) the proportion of symptoms showing significant intercept differences (out of 20 possible symptoms), and (2) the proportion of partial associations showing significant differences (out of 380 possible co-occurrences). For each ethnicity, individual intercept and partial association group differences were visualised using heatmap plots, with detailed two-way and three-way interaction values available in Supplementary material 1. Positive values indicate stronger effects (higher intercepts or stronger partial associations) in the reference group, while negative values indicate stronger effects in the comparison group. All materials, including individual symptom network plots, have been made publicly available via the Open Science Framework [49] and can be accessed at https://osf.io/k4g79/.

## Results

### Demographics

In the analysed sample (*n* = 147,037), White British was the largest ethnic category (*n* = 65,138; 44.3%) and the sample was predominantly female (98,926; 67.3%); the smallest ethnic category was Black Other (*n* = 2747; 1.87%). Across the ethnic categories, most individuals were being treated for depression (*n* = 60,372; 41.1%) followed by generalised anxiety disorder (GAD) (*n* = 19,569; 13.3%), but problem specifications were missing for 27.7% (*n* = 40,770). These figures reflect the distribution of recorded problem-type categories (e.g. Depression, GAD, Mixed Anxiety and Depression, obsessive–compulsive disorder; OCD, Phobias, post-traumatic stress disorder; PTSD, Social Phobia, Unspecified Anxiety) within this assessed cohort, rather than prevalence across the full range of mental health presentations (Table 2).

**Table 2:** Descriptive data for sample

	White British	White Irish	White other	Mixed	Indian	Pakistani	Bangladeshi	Asian	Black African	Black Caribbean	Black other	Overall
	(*n* = 65,138)	(*n* = 3,994)	(*n* = 29,260)	(*n* = 10,054)	(*n* = 6,602)	(*n* = 3,449)	(*n* = 3,191)	(*n* = 6,363)	(*n* = 7,458)	(*n* = 8,421)	(*n* = 2,747)	(*n* = 147,037)
**Problem Descriptor**												
Depression	24,925 (38.3%)	1432 (35.9%)	10,932 (36.9%)	4046 (40.2%)	3761 (57.0%)	2255 (65.4%)	1620 (50.8%)	3043 (47.8%)	3175 (42.6%)	3840 (45.6%)	1343 (48.9%)	60,372 (41.1%)
GAD	10,025 (15.4%)	598 (15.0%)	4299 (14.5%)	1189 (11.8%)	639 (9.7%)	224 (6.5%)	244 (7.6%)	618 (9.7%)	704 (9.4%)	780 (9.3%)	249 (9.1%)	19,569 (13.3%)
Mixed A + D	3558 (5.5%)	249 (6.2%)	1930 (6.5%)	552 (5.5%)	287 (4.3%)	111 (3.2%)	123 (3.9%)	282 (4.4%)	397 (5.3%)	505 (6.0%)	171 (6.2%)	8165 (5.6%)
OCD	1301 (2.0%)	60 (1.5%)	407 (1.4%)	149 (1.5%)	79 (1.2%)	42 (1.2%)	48 (1.5%)	69 (1.1%)	59 (0.8%)	69 (0.8%)	26 (0.9%)	2309 (1.6%)
Phobia	3270 (5.0%)	158 (4.0%)	1737 (5.9%)	409 (4.1%)	161 (2.4%)	101 (2.9%)	105 (3.3%)	176 (2.8%)	264 (3.5%)	297 (3.5%)	86 (3.1%)	6764 (4.6%)
PTSD	1314 (2.0%)	130 (3.3%)	1151 (3.9%)	368 (3.7%)	137 (2.1%)	95 (2.8%)	77 (2.4%)	283 (4.4%)	468 (6.3%)	388 (4.6%)	121 (4.4%)	4532 (3.1%)
Social Phobia	1844 (2.8%)	121 (3.0%)	596 (2.0%)	278 (2.8%)	89 (1.3%)	39 (1.1%)	44 (1.4%)	115 (1.8%)	186 (2.5%)	169 (2.0%)	57 (2.1%)	3538 (2.4%)
Unspec. Anx	417 (0.6%)	13 (0.3%)	97 (0.3%)	39 (0.4%)	135 (2.0%)	105 (3.0%)	80 (2.5%)	61 (1.0%)	24 (0.3%)	32 (0.4%)	15 (0.5%)	1018 (0.7%)
Missing	18,484 (28.4%)	1233 (30.9%)	8471 (28.6%)	3024 (30.1%)	1314 (19.9%)	477 (13.8%)	850 (26.6%)	1716 (27.0%)	2181 (29.2%)	2341 (27.8%)	679 (24.7%)	40,770 (27.7%)
**Gender**												
Female	41,650 (63.9%)	2562 (64.1%)	21,305 (71.9%)	7204 (71.7%)	4352 (65.9%)	2113 (61.3%)	2079 (65.2%)	4209 (66.1%)	5218 (70.0%)	6258 (74.3%)	1976 (71.9%)	98,926 (67.3%)
Male	23,488 (36.1%)	1432 (35.9%)	8315 (28.1%)	2850 (28.3%)	2250 (34.1%)	1336 (38.7%)	1112 (34.8%)	2154 (33.9%)	2240 (30.0%)	2163 (25.7%)	771 (28.1%)	48,111 (23.7%)
Age	38.4 (14.6)	41.9 (15.1)	37.0 (11.9)	33.1 (11.5)	39.0 (14.0)	35.5 (11.7)	33.4 (11.2)	36.5 (13.0)	35.5 (12.5)	39.5 (14.0)	36.5 (12.5)	37.5 (13.7)
PHQ-9 (1)	1.29 (1.01)	1.39 (1.06)	1.46 (1.04)	1.47 (1.02)	1.45 (1.01)	1.65 (1.02)	1.65 (1.03)	1.58 (1.04)	1.58 (1.07)	1.51 (1.04)	1.54 (1.06)	1.41 (1.03)
PHQ-9 (2)	1.79 (0.97)	1.81 (0.99)	1.92 (0.97)	1.95 (0.95)	1.96 (0.97)	2.16 (0.94)	2.14 (0.95)	2.03 (0.96)	2.04 (0.99)	2.01 (0.96)	2.06 (0.95)	1.89 (0.97)
PHQ-9 (3)	1.94 (1.06)	2.01 (1.06)	1.98 (1.06)	2.07 (1.03)	2.04 (1.05)	2.15 (1.02)	2.15 (1.03)	2.06 (1.04)	2.10 (1.05)	2.15 (1.03)	2.20 (1.02)	2.00 (1.06)
PHQ-9 (4)	2.00 (0.98)	2.02 (1.0)	2.10 (0.961)	2.11 (0.96)	2.09 (0.97)	2.24 (0.94)	2.27 (0.92)	2.17 (0.93)	2.11 (0.98)	2.13 (0.96)	2.16 (0.95)	2.07 (0.97)
PHQ-9 (5)	1.43 (1.14)	1.52 (1.16)	1.54 (1.13)	1.67 (1.12)	1.64 (1.12)	1.80 (1.12)	1.79 (1.10)	1.65 (1.12)	1.82 (1.11)	1.80 (1.11)	1.87 (1.09)	1.56 (1.14)
PHQ-9 (6)	1.83 (1.06)	1.89 (1.06)	1.87 (1.06)	1.96 (1.03)	1.93 (1.07)	2.00 (1.06)	2.04 (1.05)	2.01 (1.06)	1.98 (1.09)	1.94 (1.09)	2.03 (1.04)	1.89 (1.06)
PHQ-9 (7)	1.56 (1.08)	1.63 (1.09)	1.70 (1.10)	1.68 (1.08)	1.70 (1.08)	1.87 (1.09)	1.87 (1.08)	1.80 (1.08)	1.75 (1.11)	1.64 (1.10)	1.72 (1.08)	1.65 (1.09)
PHQ-9 (8)	0.90 (1.03)	1.00 (1.10)	1.08 (1.10)	1.03 (1.07)	1.15 (1.10)	1.36 (1.13)	1.30 (1.11)	1.22 (1.11)	1.15 (1.12)	1.05 (1.10)	1.12 (1.11)	1.02 (1.08)
PHQ-9 (9)	0.58 (0.86)	0.60 (0.90)	0.61 (0.90)	0.68 (0.91)	0.68 (0.95)	0.76 (1.01)	0.73 (0.95)	0.78 (0.99)	0.70 (0.97)	0.67 (0.93)	0.71 (0.96)	0.63 (0.90)
GAD-7 (1)	2.11 (0.94)	2.13 (0.94)	2.14 (0.92)	2.14 (0.93)	2.09 (0.96)	2.16 (0.94)	2.17 (0.96)	2.12 (0.94)	2.07 (0.99)	2.01 (0.99)	2.11 (0.97)	2.11 (0.94)
GAD-7 (2)	2.05 (0.99)	2.08 (0.99)	2.11 (0.97)	2.14 (0.96)	2.20 (0.95)	2.33 (0.99)	2.30 (0.91)	2.22 (0.95)	2.19 (0.97)	2.16 (0.98)	2.22 (0.95)	2.11 (0.98)
GAD-7 (3)	2.12 (0.97)	2.16 (0.96)	2.20 (0.94)	2.23 (0.93)	2.22 (0.95)	2.35 (0.91)	2.31 (0.92)	2.27 (0.94)	2.30 (0.94)	2.27 (0.94)	2.32 (0.93)	2.18 (0.95)
GAD-7 (4)	1.92 (1.00)	1.95 (1.01)	2.03 (0.98)	2.00 (0.99)	2.04 (0.98)	2.12 (0.96)	2.09 (0.98)	2.03 (1.0)	1.97 (1.03)	1.94 (1.03)	2.03 (1.00)	1.97 (1.00)
GAD-7 (5)	1.11 (1.08)	1.17 (1.11)	1.24 (1.11)	1.20 (1.10)	1.31 (1.11)	1.46 (1.14)	1.43 (1.13)	1.36 (1.12)	1.24 (1.12)	1.17 (1.10)	1.25 (1.11)	1.19 (1.10)
GAD-7 (6)	1.72 (1.02)	1.70 (1.03)	1.82 (1.03)	1.88 (1.02)	1.83 (1.04)	1.97 (1.03)	1.96 (1.00)	1.89 (1.02)	1.82 (1.08)	1.87 (1.05)	1.92 (1.06)	1.79 (1.03)
GAD-7 (7)	1.51 (1.13)	1.61 (1.13)	1.70 (1.12)	1.63 (1.12)	1.72 (1.12)	1.97 (1.09)	1.91 (1.09)	1.85 (1.11)	1.83 (1.13)	1.66 (1.14)	1.77 (1.13)	1.63 (1.13)
WSAS (2)	3.29 (2.39)	3.54 (2.48)	3.60 (2.52)	3.61 (2.49)	3.67 (2.52)	4.03 (2.58)	3.90 (2.57)	3.78 (2.58)	3.83 (2.65)	3.81 (2.60)	3.86 (2.60)	3.52 (2.49)
WSAS (3)	4.01 (2.44)	4.29 (2.54)	4.30 (2.56)	4.40 (2.51)	4.29 (2.59)	4.60 (2.69)	4.40 (2.65)	4.38 (2.63)	4.59 (2.73)	4.54 (2.69)	4.66 (2.64)	4.23 (2.54)
WSAS (4)	3.31 (2.51)	3.68 (2.60)	3.77 (2.64)	3.72 (2.62)	3.82 (2.66)	4.20 (2.73)	3.99 (2.71)	3.86 (2.71)	3.94 (2.76)	3.83 (2.75)	3.92 (2.76)	3.59 (2.62)
WSAS (5)	3.79 (2.46)	3.90 (2.52)	3.99 (2.51)	4.24 (2.52)	4.23 (2.60)	4.44 (2.67)	4.20 (2.63)	4.17 (2.59)	4.24 (2.69)	4.26 (2.65)	4.40 (2.61)	3.99 (2.53)

Notes. Mixed A + D = mixed anxiety and depression, OCD = obsessive–compulsive disorder, PTSD = post-traumatic stress disorder

### Stability testing of the results

In 100 permutations that sought to refit the 11 MNMs to produce a sampling distribution under the null hypothesis of no group differences, no significant differences were identified in the proportion of intercepts or partial associations between the observed ethnicity and, independently, the remaining ethnicities in the dataset. No obtained significant differences in the permutations indicate that the observed data do not significantly deviate from what would be expected by chance. This suggests an absence of meaningful variation in the permutated data metrics across ethnic groups, indicating stability in the observed results. The absence of variation in the results implies that, assuming no real differences exist, false positives are rare within this dataset and so the observed results are stable across 100 permutations. Chi-square goodness-of-fit test (*χ*^2^(10) = 10.068, *p* = 0.4346) indicated that there were no statistically significant differences in the distribution of counts (average number of estimated edges) across the 11 ethnic groups.

### Differences in individual symptoms and their co-occurrences among ethnic groups

Out of 55 pairwise, network level, ethnic comparisons (the intercepts), there were 48 (89.1%) significant differences identified (Table 3). Of these symptom-level differences, four between-ethnicity differences within the generated networks were observed to be ≥50% in all the possible comparisons, controlling for the influence of age, gender, and all other symptoms (White British x Black Caribbean = 0.75, White British x Black African = 0.70, White other x Black African = 0.50 and White other x Black Caribbean = 0.5). Overall, White British had the most symptom differences with all other ethnicities [76], with the largest number of differences arising in anxiety-related symptoms (e.g. nervousness, excessive worry) and functional impairment items. This was then followed by White other [64] , Black Caribbean [58], Black African [48], Indian [40], Pakistani [38], Mixed [36], White Irish [36], Asian [32], Bangladeshi [29] and Black other [17]. When changing the reference ethnicity, the results were broadly similar and are reported in Supplementary material 2: Table S3 and Supplementary material 2: Table S4.

**Table 3:** Proportion of significant group differences for individual symptoms (nodes)

	1	2	3	4	5	6	7	8	9	10	11
1. White British		0.05	0.5	0.25	0.4	0.4	0.3	0.35	0.6	0.75	0.2
2. White Irish			0.1	0.1	0.2	0.25	0.15	0.25	0.35	0.2	0.15
3. White other				0.35	0.25	0.35	0.25	0.25	0.45	0.5	0.2
4. Mixed					0	0.35	0.2	0.15	0.25	0.1	0.05
5. Indian						0.1	0.2	0.1	0.3	0.3	0.1
6. Pakistani							0	0	0.05	0.3	0
7. Bangladeshi								0	0.05	0.3	0.05
8. Asian-other-or-Chinese									0.15	0.3	0.1
9. Black African										0.2	0
10. Black Caribbean											0
11. Black other											

Notes. These are significant group differences between the reference group (y axis) and the comparative ethnicity (x axis), indicating a difference in the strength of endorsement for a given symptom; the two-way interactions. The value is a total proportion of detected differences out of 20 available symptoms (nine PHQ-9 symptoms, seven GAD-7 symptoms and four WSAS indicators)

To aid interpretation, differences shown in the heatmaps indicate where two ethnic groups differ in the strength of endorsement for a given symptom. The colour intensity reflects the magnitude of the difference, with red indicating higher scores for the first group in the pair and blue indicating higher scores for the second. The tables summarise these findings by reporting the proportion of symptoms or symptom pairs that differ between groups, allowing readers to compare the extent of differences across ethnicities.

**Fig. 1:**
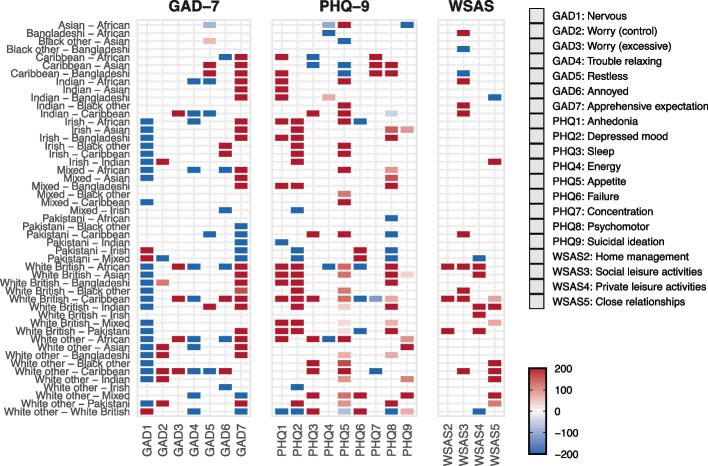
Heatmap plot displaying significant differences in symptom expression across ethnic groups for assessment scales: PHQ-9, GAD-7, and WSAS.* Notes.* Values represent percentage absolute mean differences between symptom intercepts in pairwise ethnic group comparisons (range: −200% to 200%), with larger absolute values indicating greater differences; extreme values (≥100%) indicate that one group’s estimate is substantially larger or close to zero relative to the other. Red indicates higher symptom levels in the first group listed, and blue indicates higher levels in the second group. Only statistically significant differences are shown

Figure 1 presents a heatmap plot visualising significant pairwise, network-level ethnic comparisons of symptom differences for the PHQ-9, GAD-7, and WSAS scales (specific values are included in the supplement). In each plot, the symptoms measured by the respective scale are displayed along the horizontal axis, and the ethnic group comparisons are shown vertically. In networks, an edge refers to the statistical association between two symptoms after controlling for all others, and differences in edges indicate where these symptom-to-symptom relationships vary across ethnicities. The connecting edges between symptoms indicate significant differences in symptom expression between ethnic groups. The colour gradient represents the magnitude and direction of the difference between groups: red indicates that the first group listed in the pair scored significantly higher than the second, whereas blue indicates the first group scored significantly lower. Only statistically significant differences are displayed. This visualisation allows for a clear overview of how symptom patterns differ across ethnic groups, highlighting areas where differences in symptom expression are most pronounced. By controlling for the influence of other symptoms, these plots provide insight into the unique contributions of each symptom to ethnic differences in mental health presentations.

For the PHQ-9, which measures depression symptoms, the most pronounced between-ethnicity differences were found in item 2 (feeling down or hopeless), with 9 (out of 55 possible comparisons between 11 ethnicities) significant differences observed. Items 1 (little interest or pleasure in activities), 5 (poor appetite or overeating), and 8 (moving/speaking slowly or being fidgety/restless) each showed 7 differences. Items 3 (trouble sleeping or sleeping too much), 6 (feeling bad about yourself), and 9 (thoughts of self-harm or being better off dead) displayed 5 differences each. The least variation was seen in items 4 (feeling tired or having little energy) and 7 (trouble concentrating), with 2 and 1 differences, respectively.

The GAD-7, assessing anxiety symptoms, revealed the highest number of between-ethnicity differences (9 each) for items 1 (feeling nervous, anxious, or on edge) and 7 (feeling afraid something awful might happen). Items 2 (unable to stop or control worrying) and 4 (trouble relaxing) both showed 5 differences. Items 5 (being so restless it is hard to sit still) and 6 (becoming easily annoyed or irritable) each had 4 differences, while item 3 (worrying too much about different things) showed the least variation with 2 differences.

The WSAS, which evaluates work and social adjustment, demonstrated the most between-ethnicity differences in items 4 (private leisure activities impairment) and 5 (ability to form/maintain relationships impairment), with 6 differences each. Item 3 (home management impairment) showed 3 differences, and item 2 (work impairment) revealed 2 differences.

**Fig. 2:**
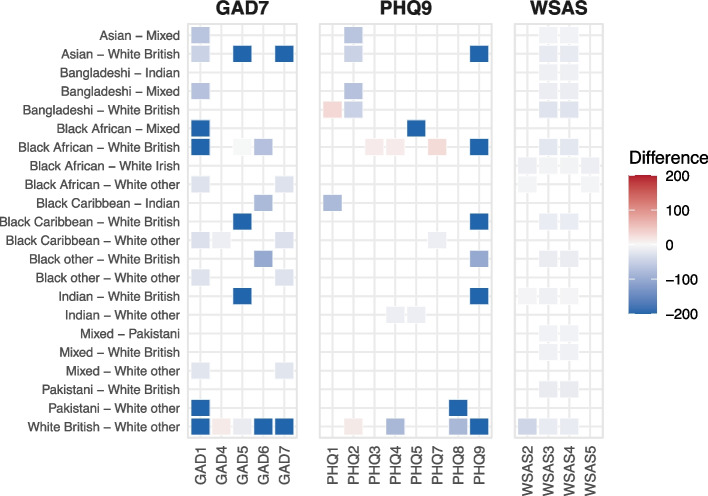
Heatmap plot showing significant differences in symptom co-occurrence between ethnic groups.* Notes.* Values represent percentage absolute mean differences between partial associations in pairwise ethnic group comparisons (range: −200% to 200%), with larger absolute values indicating greater differences; extreme values (≥100%) indicate that one group’s estimate is substantially larger or close to zero relative to the other. Red indicates stronger associations in the first group listed, and blue indicates stronger associations in the second group. Only statistically significant differences are shown

Figure 2 presents a heatmap plot visualising significant differences in symptom co-occurrence between ethnic groups. There were 22 out of a possible 55 partial association differences (the strength in relationship between two nodes) between 20 ethnicity pairs. These ranged from 1 to 5%, with the greatest proportion of differences being identified between White British and White Other (Table 4). There were 14 comparatively small differences between ethnicities in the relationship between impairment in home management and impairment in private leisure activities. There were 5 comparatively large differences, reflecting the presence vs. an absence for the co-occurrence between restless and suicidal ideation (GAD-7 Q5 and PHQ-9 Q9). The association was present in the White British network but was not observed in the White Other, Asian, Black Caribbean, Indian, and Black African networks.

**Table 4:** Proportion of significant group differences for symptom co-occurrence (edges)

	1	2	3	4	5	6	7	8	9	10	11
1. White British		0	0.05	0.01	0.02	0.01	0.02	0.02	0.04	0.01	0.01
2. White Irish			0	0	0	0	0	0	0.01	0	0
3. White other				0.01	0.01	0.01	0	0	0.01	0.01	0.01
4. Mixed					0	0.01	0.01	0.01	0.01	0	0
5. Indian						0	0.01	0	0	0.01	0
6. Pakistani							0	0	0	0	0
7. Bangladeshi								0	0	0	0
8. Asian-other-or-Chinese									0	0	0
9. Black African										0	0
10. Black Caribbean											0
11. Black other											

Notes. These are significant group differences between the reference group and the comparative ethnicity, indicating a difference in the strength of the relationship between two symptoms; the three-way interactions. The value is a total proportion of detected differences out of 380 possible symptom co-occurrences (20 available symptoms with 19 possible connections)

## Discussion

This study examined differences in how symptoms of anxiety and depression, along with social functioning, are expressed and relate to each other across ethnic groups. A network analysis of psychological symptoms in individuals seeking psychological support was conducted, moderated by ethnicity and controlled for age and gender. The study found a high proportion of significant differences in network-level comparisons across ethnicities, indicating substantial variation in symptom networks among different ethnic groups. Ethnicity was associated with the strength of symptom endorsement, but once endorsed, symptom co-occurrence differences between ethnicities were limited. The results suggest that patterns of symptom expression may vary across ethnic groups, which could have significant implications for assessment and treatment approaches in diverse populations.

This large-scale, well-powered analysis uses ethnic categories, and the observed differences may reflect both underlying variation in symptom expression and the limitations of these classifications. These categories are proxies for complex cultural, social, and historical factors, and therefore cannot capture the full range of influences on symptom expression [32]. Consequently, caution should be exercised when interpreting the results, as the findings reflect broad patterns and may not capture the full range of cultural variation within each ethnic group.

White British individuals, who are in a majority in the UK population, showed the most differences when compared to all other ethnicities. The substantial differences observed, particularly between the White British and Black groups, suggest that ethnicity-related factors (including cultural, socioeconomic, and structural influences) may play a significant role in symptom expression. These differences may also reflect broader factors, including socioeconomic position, structural inequalities, and differences in access to resources, which were not directly measured in this study. Consequently, examining population data without reference to ethnicity may obscure important differences among ethnic groups. The overrepresentation of White British participants in many studies, including most clinical trials [50], may lead to biased understanding of mental health symptoms and experiences. This could result in diagnostic criteria and treatment approaches that are not equally effective or suitable for all ethnic groups. These findings highlight the importance of recognising that symptom expression may vary across ethnic groups, and that assessment should consider how different symptoms may be more or less prominently reported across groups.

The findings reveal significant variability in the manifestation and reporting of psychological symptoms and functioning across ethnicities, emphasising the importance of cultural considerations in mental health assessment and treatment. Depressive symptoms, such as anhedonia and low mood, as well as anxiety-related symptoms, such as nervousness and catastrophic thinking (individually and co-occurring), showed substantial variations among different ethnic groups, with some groups (e.g. all categories except White British, Irish, Indian or Black Other for anhedonia, or all categories except White British and Irish for nervousness) endorsing them much less frequently than others, potentially leading to misdiagnosis and inappropriate understandings of recovery within therapy. Appetite and psychomotor activity also demonstrated variability, though the nature of these differences warrants further investigation due to item wording. Cultural variation in functioning was observed in leisure activities and relationship impairment, and the relationship between home management and private leisure activities impairment, indicating that cultural factors influence these domains.

Symptom co-occurrence was less variable, but the most differences were observed between White British and White Other groups, particularly in anxiety-related symptoms and functional impairment. For instance, the edge between nervousness (item 1) and irritability (item 6) was present for White British but not for White Other participants. Similar distinctions were found between Black African and White British groups, such as the relationship between restlessness (item 5) and suicidal ideation (item 9), which was absent in the Black African group and aligns with findings in an African (Ethiopian) depression network study [51].

Despite the observed variability, the study identified several consistencies across ethnic groups. Symptoms like concentration difficulties and low energy appeared to be universally experienced across cultures. Excessive worry emerged as a consistent aspect of anxiety across ethnicities. In the analysis of symptom co-occurrence, fewer differences were observed across ethnicities compared to individual symptom variations, indicating that ethnicity has a less substantial impact on these higher-order relationships. However, this observation should be interpreted cautiously, as the regularisation procedure employed to prevent overfitting may have suppressed weaker moderation effects. No ethnic differences were observed in the relationship between psychomotor agitation and suicidal ideation, in line with similar network studies [51, 52]. This consistency is particularly noteworthy given that agitation is the most frequently reported symptom among individuals with a history of suicide attempts [53, 54].

The finding that anxiety and depression symptoms are differentially endorsed across different ethnic groups highlights the risk of relying on symptoms lists derived from predominantly White, American individuals in the United States. Relying on measures developed within this narrow cultural context means that culturally specific presentations may be overlooked or misinterpreted. For example, previous cross-cultural research indicates that symptoms such as loneliness are central to depression in some African populations yet are not emphasised in standard criteria based on White British groups [5]. Such discrepancies raise the risk of misdiagnosis, as presentations falling outside standard assessment frameworks may be under-recognised, potentially skewing diagnostic decisions and the interpretation of clinical progress.

Consequently, these findings offer a practical framework for improving cultural competence in assessment [55]. Rather than treating group-level data as a rigid diagnostic rule, which would be inappropriate given significant within-group heterogeneity, clinicians should use these patterns as a heuristic for inquiry. This approach allows clinicians to anticipate and actively probe for difficulties that patients from different backgrounds may prioritise (e.g., somatic nervousness vs. cognitive worry), thereby ensuring the assessment captures the full picture of a patient’s distress. To support this, future assessment approaches must be more comprehensive, potentially using qualitative methods to explore the culturally shaped meanings associated with specific symptoms.

Regarding intervention, these distinct symptom profiles suggest that cultural adaptation may also involve consideration of symptom content, rather than implementation alone. Interventions should be tailored to address the symptoms rather than applying a generic cognitive model. By validating the patient’s specific idiom of distress, clinicians can enhance the therapeutic alliance and address poor engagement rates observed in minoritised groups [56]. Furthermore, measuring treatment change must account for these differences to ensure that progress is defined by the reduction of symptoms relevant to the patient’s cultural experience. These findings are not intended to replace standardised measures, but to support their more informed use within existing frameworks. Future research should prioritize documenting these content-specific adaptations and evaluating their impact on diverse groups [21].

The ONS standardised ethnic measure enables cross-study comparability [32], though these broad categories mask significant within-group diversity. Data such as first language, generation, and religion would provide more context around ethnicity. It is acknowledged that selection biases possibly exist in the sample, particularly in individuals who seek and obtain care, as well as those who complete psychological measures. Although a substantial proportion of the original sample was excluded due to incomplete item-level data, the missingness did not follow any notable demographic or clinical pattern, reducing the sample size but not indicating systematic bias across ethnic groups. Despite the large sample size, smaller ethnic groups were combined to ensure statistical comparison. While aggregation risks overlooking individual cultural identities and intra-group variations, excluding these groups entirely would have prevented examination of their unique perspectives. It is important to contextualise these findings within the specific sociopolitical landscape of the United Kingdom. The ethnic categories used in this study reflect the history of migration and identity in the UK and align with standard UK Census classifications. As definitions of race and ethnicity are socially constructed and vary globally, these specific groupings may not map directly onto categorization systems used in other jurisdictions. Therefore, while our findings highlight the value of granular analysis, the specific ethnic profiles reported here should be interpreted within the UK context. We suggest that researchers in other jurisdictions replicate this approach using ethnic taxonomies that reflect their own local demographic realities.

The study establishes a foundation for examining culture-symptomology relationships, despite its limitations. It offers a broad perspective that can guide more focused investigations in the future. While subsequent studies may lack the statistical power of this large-scale analysis, they will be crucial in unpacking the complex cultural factors that our proxy description of ethnicity cannot fully capture. Studies using large-scale datasets in the future should consider how the distribution of ethnic groups within a sample may influence the apparent stability of individual symptom items, and where possible, examine item-level patterns across groups rather than relying solely on total scores. Future studies should also develop and utilise more refined measures of cultural identity and experience to build upon insights from this broader approach [37].

It is important to acknowledge the transdiagnostic nature of the symptoms measured [27] with significant symptom overlap between disorders [57]. The observed group differences may partly reflect variation in co-occurring difficulties rather than depression and anxiety. We note that node exclusion can affect network estimation [58, 59]. However, in this study the exclusion of WSAS item 1 was necessary due to its non-applicability for a large proportion of participants and including it would have introduced greater bias by disproportionately reducing the analytic sample. Further research should explore the phenomenology of symptom expression across ethnic groups to understand how cultural, social, and psychological factors shape the experience and expression of mental health symptoms. This approach will help identify how symptoms are perceived, described, and co-occur in different populations, ensuring more accurate diagnoses [60] and culturally sensitive interventions.

## Conclusions

This study, conducted in the context of London, United Kingdom, highlighted significant ethnic variations in symptom networks, with differences in individual symptoms and smaller variations in symptom co-occurrence across the observed ethnic groups. These findings underscore the role of cultural competence in mental health assessment and treatment, highlighting the need for a nuanced understanding of how symptom expression and their impacts differ across ethnic groups.

## Supplementary Information

Supplementary Material 1. Table S1. Pairwise ethnic group differences in symptom expression (intercepts; two-way interactions). Description: Contains detailed values of significant differences in symptom intercepts across all pairwise ethnic group comparisons. Table S2: Pairwise ethnic group differences in symptom partial associations (three-way interactions).

Supplementary Material 2. Table S3. Pairwise ethnic group differences in symptom expression with rotated reference groups (two-way interactions). Description: Provides sensitivity analyses showing intercept differences when the reference ethnicity is systematically rotated across models. Table S4. Pairwise ethnic group differences in symptom co-occurrence with rotated reference groups (three-way interactions). Description: Provides sensitivity analyses showing partial associations differences when the reference ethnicity is systematically rotated across models.

Supplementary Material 3. Analysis code for moderated network models. Description: R code used to prepare the dataset, estimate moderated network models, compute intercept and edge differences, and perform permutation testing.

## Data Availability

The data used in this study are not publicly available due to the use of sensitive patient data from NHS services. However, data access requests can be made to the corresponding author, subject to appropriate governance approvals.

## References

[1] Murray CJL. The Global Burden of Disease Study at 30 years. Nat Med. 2022;28(10):2019–26. 10.1038/s41591-022-01990-1. 36216939 10.1038/s41591-022-01990-1

[2] Chisholm D, Sweeny K, Sheehan P, Rasmussen B, Smit F, Cuijpers P, et al. Scaling-up treatment of depression and anxiety: a global return on investment analysis. Lancet Psychiatry. 2016;3(5):415–24. 10.1016/S2215-0366(16)30024-4. 27083119 10.1016/S2215-0366(16)30024-4

[3] Fried EI, Flake JK, Robinaugh DJ. Revisiting the theoretical and methodological foundations of depression measurement. Nat Rev Psychol. 2022. 10.1038/s44159-022-00050-2. 38107751 10.1038/s44159-022-00050-2 PMC10723193

[4] Dere J, Sun J, Zhao Y, Persson TJ, Zhu X, Yao S, et al. Beyond “somatization” and “psychologization”: symptom-level variation in depressed Han Chinese and Euro-Canadian outpatients. Front Psychol. 2013;4. 10.3389/fpsyg.2013.00377 10.3389/fpsyg.2013.00377 PMC3694214 23818884

[5] Haroz EE, Ritchey M, Bass JK, Kohrt BA, Augustinavicius J, Michalopoulos L, et al. How is depression experienced around the world? A systematic review of qualitative literature. Soc Sci Med. 2017;183:151–62. 10.1016/j.socscimed.2016.12.030. 28069271 10.1016/j.socscimed.2016.12.030 PMC5488686

[6] Bass JK, Bolton PA, Murray LK. Do not forget culture when studying mental health. Lancet. 2007;370(9591):918–9. 10.1016/S0140-6736(07)61426-3. 17869621 10.1016/S0140-6736(07)61426-3

[7] NCCMH. Ethnic Inequalities in Improving Access to Psychological Therapies (IAPT). UK: NHS Race and Health Observatory; 2023. Report No. Available from: https://www.nhsrho.org/wp-content/uploads/2023/10/Ethnic-Inequalities-in-Improving-Access-to-Psychological-Therapies-IAPT.Full-report.pdf

[8] Harwood H, Rhead R, Chui Z, Bakolis I, Connor L, Gazard B, et al. Variations by ethnicity in referral and treatment pathways for IAPT service users in South London. Psychol Med. 2023;53(3):1084–95. 10.1017/S0033291721002518. 34334151 10.1017/S0033291721002518 PMC9976018

[9] Barnett P, Mackay E, Matthews H, Gate R, Greenwood H, Ariyo K, et al. Ethnic variations in compulsory detention under the Mental Health Act: a systematic review and meta-analysis of international data. Lancet Psychiatry. 2019;6(4):305–17. 10.1016/S2215-0366(19)30027-6. 30846354 10.1016/S2215-0366(19)30027-6 PMC6494977

[10] Bhavsar V, Jannesari S, McGuire P, MacCabe JH, Das-Munshi J, Bhugra D, et al. The association of migration and ethnicity with use of the Improving Access to Psychological Treatment (IAPT) programme: a general population cohort study. Soc Psychiatry Psychiatr Epidemiol. 2021;56(11):1943–56. 10.1007/s00127-021-02035-7. 33591376 10.1007/s00127-021-02035-7 PMC8519879

[11] Amati F, Green J, Kitchin L, Watt H, Jones S, AlRubaye N, et al. Ethnicity as a predictor of outcomes of psychological therapies for anxiety and depression: a retrospective cohort analysis. Behav Cogn Psychother. 2023;51(2):164–73. 10.1017/S1352465822000558. 36740941 10.1017/S1352465822000558

[12] Arundell LLC, Saunders R, Buckman JEJ, Lewis G, Stott J, Singh S, et al. Differences in psychological treatment outcomes by ethnicity and gender: an analysis of individual patient data. Soc Psychiatry Psychiatr Epidemiol. 2024. 10.1007/s00127-024-02610-8. 38321296 10.1007/s00127-024-02610-8 PMC11343885

[13] Buckman JEJ, Saunders R, Cape J, Pilling S. Establishing a service improvement network to increase access to care and improve treatment outcomes in community mental health: a series of retrospective cohort studies. Lancet. 2021;398:S28. 10.1016/S0140-6736(21)02571-X.34227960

[14] Karasz A, Gany F, Escobar J, Flores C, Prasad L, Inman A, et al. Mental Health and Stress Among South Asians. J Immigr Minor Health. 2019;21(S1):7–14. 10.1007/s10903-016-0501-4. 27848078 10.1007/s10903-016-0501-4 PMC5643212

[15] Causadias JM. What is culture? Systems of people, places, and practices. Appl Dev Sci. 2020;24(4):310–22. 10.1080/10888691.2020.1789360.

[16] Marsella AJ, Yamada AM. Culture and Psychopathology: Foundations, Issues, Directions. J Pac Rim Psychol. 2010;4(2):103–15. 10.1375/prp.4.2.103.

[17] Whaley AL, Davis KE. Cultural competence and evidence-based practice in mental health services: A complementary perspective. Am Psychol. 2007;62(6):563–74. 10.1037/0003-066X.62.6.563. 17874897 10.1037/0003-066X.62.6.563

[18] Arundell LL, Barnett P, Buckman JEJ, Saunders R, Pilling S. The effectiveness of adapted psychological interventions for people from ethnic minority groups: a systematic review and conceptual typology. Clin Psychol Rev. 2021;88:102063. 10.1016/j.cpr.2021.102063. 34265501 10.1016/j.cpr.2021.102063 PMC8591374

[19] Griner D, Smith TB. Culturally adapted mental health intervention: a meta-analytic review. Psychother Theory Res Pract Train. 2006;43(4):531–48. 10.1037/0033-3204.43.4.531. 10.1037/0033-3204.43.4.531 22122142

[20] Chowdhary N, Jotheeswaran AT, Nadkarni A, Hollon SD, King M, Jordans MJD, et al. The methods and outcomes of cultural adaptations of psychological treatments for depressive disorders: a systematic review. Psychol Med. 2014;44(6):1131–46. 10.1017/S0033291713001785. 23866176 10.1017/S0033291713001785 PMC3943384

[21] Soto A, Smith TB, Griner D, Domenech Rodríguez M, Bernal G. Cultural adaptations and therapist multicultural competence: two meta‐analytic reviews. J Clin Psychol. 2018;74(11):1907–23. 10.1002/jclp.22679. 30091201 10.1002/jclp.22679

[22] Benuto LT, Casas J, O’Donohue WT. Training culturally competent psychologists: a systematic review of the training outcome literature. Train Educ Prof Psychol. 2018;12(3):125–34. 10.1037/tep0000190.

[23] Benuto LT, O’Donohue W. Is culturally sensitive cognitive behavioral therapy an empirically supported treatment?: The case for Hispanics. Int J Psychol Psychol Ther. 2015;15(3):405–21.

[24] Huey SJ, Polo AJ. Evidence-based psychosocial treatments for ethnic minority youth. J Clin Child Adolesc Psychol. 2008;37(1):262–301. 10.1080/15374410701820174. 18444061 10.1080/15374410701820174 PMC2413000

[25] Kim MT, Heitkemper EM, Hébert ET, Hecht J, Crawford A, Nnaka T, et al. Redesigning culturally tailored intervention in the precision health era: self-management science context. Nurs Outlook. 2022;70(5):710–24. 10.1016/j.outlook.2022.05.015. 35933178 10.1016/j.outlook.2022.05.015 PMC9722518

[26] Hofmann SG, Hinton DE. Cross-cultural aspects of anxiety disorders. Curr Psychiatry Rep. 2014;16(6):450. 10.1007/s11920-014-0450-3. 24744049 10.1007/s11920-014-0450-3 PMC4037698

[27] O’Driscoll C, Buckman JEJ, Fried EI, Saunders R, Cohen ZD, Ambler G, et al. The importance of transdiagnostic symptom level assessment to understanding prognosis for depressed adults: analysis of data from six randomised control trials. BMC Med. 2021;19(1):109. 10.1186/s12916-021-01971-0. 33952286 10.1186/s12916-021-01971-0 PMC8101158

[28] De La Torre‐Luque A, Ojagbemi A, Caballero FF, Lara E, Moreno‐Agostino D, Bello T, et al. Cross‐cultural comparison of symptom networks in late‐life major depressive disorder: Yoruba Africans and the Spanish population. Int J Geriatr Psychiatry. 2020;35(9):1060–8. 10.1002/gps.5329. 32394534 10.1002/gps.5329

[29] Fonseca-Pedrero E, Ortuño J, Debbané M, Chan RCK, Cicero D, Zhang LC, et al. The network structure of schizotypal personality traits. Schizophr Bull. 2018;44(suppl_2):S468-79. 10.1093/schbul/sby044. 29684178 10.1093/schbul/sby044 PMC6188518

[30] Fried EI, Eidhof MB, Palic S, Costantini G, Huisman-van Dijk HM, Bockting CLH, et al. Replicability and generalizability of posttraumatic stress disorder (PTSD) networks: a cross-cultural multisite study of PTSD symptoms in four trauma patient samples. Clin Psychol Sci. 2018;6(3):335–51. 10.1177/2167702617745092. 29881651 10.1177/2167702617745092 PMC5974702

[31] Mihić L, Janičić B, Marchetti I, Novović Z, Sica C, Bottesi G, et al. Comorbidity among depression, anxiety and stress symptoms in naturalistic clinical samples: a cross‐cultural network analysis. Clin Psychol Psychother. 2024;31(1):e2927. 10.1002/cpp.2927. 37940606 10.1002/cpp.2927

[32] Connelly R, Gayle V, Lambert PS. Ethnicity and ethnic group measures in social survey research. Methodol Innov. 2016;9:205979911664288. 10.1177/2059799116642885.

[33] Aspinall PJ. The utility and validity for public health of ethnicity categorization in the 1991, 2001 and 2011 British Censuses. Public Health. 2011;125(10):680–7. 10.1016/j.puhe.2011.05.001. 21907364 10.1016/j.puhe.2011.05.001

[34] Office for National Statistics. Research report on population estimates by characteristics . 2017. Report No. Available from: https://www.ons.gov.uk/peoplepopulationandcommunity/populationandmigration/populationestimates/methodologies/researchreportonpopulationestimatesbycharacteristics

[35] Mohammad-Arif A. The paradox of religion: the (re)construction of Hindu and Muslim identities amongst South Asian diasporas in the United States. South Asia Multidiscip Acad J. 2007;(1). 10.4000/samaj.55

[36] Rai R, Sankaran C. Religion and the South Asian diaspora. South Asian Diaspora. 2011;3(1):5–13. 10.1080/19438192.2010.539030.

[37] Simpson L, Jivraj S, Warren J. The stability of ethnic identity in England and Wales 2001–2011. J R Stat Soc Ser A Stat Soc. 2016;179(4):1025–49. 10.1111/rssa.12175. 27773972 10.1111/rssa.12175 PMC5053233

[38] Saunders R, Cape J, Leibowitz J, Aguirre E, Jena R, Cirkovic M, et al. Improvement in IAPT outcomes over time: are they driven by changes in clinical practice? Cogn Behav Ther. 2020;13:e16. 10.1017/S1754470X20000173. 10.1017/S1754470X20000173 PMC7872157 33613689

[39] Clark DM. Realizing the mass public benefit of evidence-based psychological therapies: The IAPT program. Annu Rev Clin Psychol. 2018;14(1):1. 10.1146/annurev-clinpsy-050817-084833. 29350997 10.1146/annurev-clinpsy-050817-084833 PMC5942544

[40] Kroenke K, Spitzer RL, Williams JB. The PHQ-9: validity of a brief depression severity measure. J Gen Intern Med. 2001;16(9):9. 10.1046/j.1525-1497.2001.016009606.x. 11556941 10.1046/j.1525-1497.2001.016009606.x PMC1495268

[41] Spitzer RL, Kroenke K, Williams JBW, Löwe B. A brief measure for assessing generalized anxiety disorder: the GAD-7. Arch Intern Med. 2006;166(10):10. 10.1001/archinte.166.10.1092. 10.1001/archinte.166.10.1092 16717171

[42] Mundt JC, Marks IM, Shear MK, Greist JH. The work and social adjustment scale: a simple measure of impairment in functioning. Br J Psychiatry. 2002;180(5):461–4. 10.1192/bjp.180.5.461. 11983645 10.1192/bjp.180.5.461

[43] Saunders R, Buckman JEJ, Stott J, Leibowitz J, Aguirre E, John A, et al. Older adults respond better to psychological therapy than working-age adults: evidence from a large sample of mental health service attendees. J Affect Disord. 2021;294:85–93. 10.1016/j.jad.2021.06.084. 34274792 10.1016/j.jad.2021.06.084 PMC8411661

[44] Delamain H, Buckman JEJ, O’Driscoll C, Suh JW, Stott J, Singh S, et al. Predicting post-treatment symptom severity for adults receiving psychological therapy in routine care for generalised anxiety disorder: a machine learning approach. Psychiatry Res. 2024;336:115910. 10.1016/j.psychres.2024.115910. 38608539 10.1016/j.psychres.2024.115910

[45] Improving Access to Psychological Therapies. The IAPT Data Handbook Guidance on recording and monitoring outcomes to support local evidence-based practice. Version 2.0. 2011. Available from: http://webarchive.nationalarchives.gov.uk/20160302160058/http://www.iapt.nhs.uk/silo/files/iapt-data-handbook-v2.pdf

[46] Haslbeck JMB, Borsboom D, Waldorp LJ. Moderated network models. Multivar Behav Res. 2019;0(0):1–32. 10.1080/00273171.2019.1677207. 10.1080/00273171.2019.1677207 31782672

[47] Hastie T, Tibshirani R, Wainwright M. Statistical Learning with Sparsity: The Lasso and Generalizations. 1st edition. Boca Raton: Routledge; 2015. p. 367.

[48] Foygel R, Drton M. Extended Bayesian information criteria for Gaussian graphical models. arXiv. 2010. 10.48550/arXiv.1011.6640.

[49] Delamain H, Haslbeck JMB, Shaikh M, Buckman JEJ, Jena R, Saunders R, et al. Illuminating cultural heritage variations in mental health symptom networks. Open Science Framework; 2025. Available from: https://osf.io/k4g79/

[50] Bodicoat DH, Routen AC, Willis A, Ekezie W, Gillies C, Lawson C, et al. Promoting inclusion in clinical trials—a rapid review of the literature and recommendations for action. Trials. 2021;22(1):880. 10.1186/s13063-021-05849-7. 34863265 10.1186/s13063-021-05849-7 PMC8643184

[51] Workneh F, Worku A, Assefa N, Berhane Y. Network analysis of mental health problems among adults in Addis Ababa, Ethiopia: a community-based study during the COVID-19 pandemic. BMJ Open. 2024;14(1):e075262. 10.1136/bmjopen-2023-075262. 38253451 10.1136/bmjopen-2023-075262 PMC10806846

[52] Kivelä LMM, Fried EI, Van Der Does W, Antypa N. Examining contemporaneous and temporal associations of real-time suicidal ideation using network analysis. Psychol Med. 2024. 10.1017/s003329172400151x. 39245794 10.1017/S003329172400151X PMC11496231

[53] Ren L, Wang Y, Wu L, Wei Z, Cui LB, Wei X, et al. Network structure of depression and anxiety symptoms in Chinese female nursing students. BMC Psychiatry. 2021. 10.1186/s12888-021-03276-1. 34059013 10.1186/s12888-021-03276-1 PMC8168020

[54] Sani G, Tondo L, Koukopoulos A, Reginaldi D, Kotzalidis GD, Koukopoulos AE, et al. Suicide in a large population of former psychiatric inpatients. Psychiatry Clin Neurosci. 2011;65(3):286–95. 10.1111/j.1440-1819.2011.02205.x. 21507136 10.1111/j.1440-1819.2011.02205.x

[55] Kirmayer LJ. Cultural variations in the clinical presentation of depression and anxiety: implications for diagnosis and treatment. J Clin Psychiatry. 2001;62(suppl 13):22–8. 11434415

[56] Cooper L, Roter DL, Carson K, Beach MC, Sabin J, Greenwald AG, et al. The associations of clinicians’ implicit attitudes about race with medical visit communication and patient ratings of interpersonal care. Am J Public Health. 2012;102(5):979–87. 10.2105/AJPH.2011.300558. 22420787 10.2105/AJPH.2011.300558 PMC3483913

[57] O’Driscoll C, Epskamp S, Fried EI, Saunders R, Cardoso A, Stott J, et al. Transdiagnostic symptom dynamics during psychotherapy. Sci Rep. 2022;12(1):10881. 10.1038/s41598-022-14901-8. 35760940 10.1038/s41598-022-14901-8 PMC9237087

[58] Burger J, Isvoranu AM, Lunansky G, Haslbeck JMB, Epskamp S, Hoekstra RHA, et al. Reporting standards for psychological network analyses in cross-sectional data. Psychol Methods. 2023;28(4):806–24. 10.1037/met0000471. 35404629 10.1037/met0000471

[59] Fried EI, Cramer AOJ. Moving forward: challenges and directions for psychopathological network theory and methodology. Perspect Psychol Sci. 2017;12(6):999–1020. 10.1177/1745691617705892. 28873325 10.1177/1745691617705892

[60] Bradford A, Meyer AND, Khan S, Giardina TD, Singh H. Diagnostic error in mental health: a review. BMJ Qual Saf. 2024;33(10):663–72. 10.1136/bmjqs-2023-016996. 38575311 10.1136/bmjqs-2023-016996 PMC11503128

